# Introducing standardized “readbacks” to improve patient safety in surgery: a prospective survey in 92 providers at a public safety-net hospital

**DOI:** 10.1186/1471-2482-12-8

**Published:** 2012-06-19

**Authors:** Hari Prabhakar, Jeffrey B Cooper, Allison Sabel, Sebastian Weckbach, Philip S Mehler, Philip F Stahel

**Affiliations:** 1 Harvard Medical School, 107 Ave Louis Pasteur, Boston, MA, 02115, USA; 2 Department of Anesthesia, Critical Care, and Pain Medicine, Massachusetts General Hospital, & Center for Medical Simulation, 65 Landsdowne St., Cambridge, MA, 20139, USA; 3 Department of Biostatistics and Informatics and Department of Patient Safety and Quality, Denver Health, University of Colorado Denver, Colorado School of Public Health, 777 Bannock Street, Denver, CO, 80204, USA; 4 Department of Orthopaedics, Denver Health, University of Colorado Denver, School of Medicine, 777 Bannock Street, Denver, CO, 80204, USA; 5 Department of Patient Safety and Quality, and Department of Internal Medicine, Denver Health, University of Colorado Denver, School of Medicine, 777 Bannock Street, Denver, CO, 80204, USA; 6 Department of Orthopaedic Surgery and Department of Neurosurgery, University of Colorado, School of Medicine, Denver Health Medical Center, 777 Bannock Street, Denver, CO, 80204, USA

## Abstract

**Background:**

Communication breakdowns represent the main root cause of preventable complications which lead to harm to surgical patients. Standardized readbacks have been successfully implemented as a main pillar of professional aviation safety for decades, to ensure a safe closed-loop communication between air traffic control and individual pilots. The present study was designed to determine the perception of staff in perioperative services regarding the role of standardized readbacks for improving patient safety in surgery at a single public safety-net hospital and level 1 trauma center.

**Methods:**

A 12-item questionnaire was sent to 180 providers in perioperative services at Denver Health Medical Center. The survey was designed to determine the individual participants’ perception of (1) appropriateness of current readback processes; (2) willingness to attend a future training module on this topic; (3) specific scenarios in which readbacks may be effective; and (4) perceived major barriers to the implementation of standardized readbacks. Survey results were compared between departments (surgery versus anesthesia) and between specific staff roles (attending or midlevel provider, resident physician, nursing staff), using non-parametric tests.

**Results:**

The response rate to the survey was 50.1 % (n = 92). Respondents overwhelmingly recognized the role of readbacks in reducing communication errors and improving patient safety. There was a strong agreement among respondents to support participation in a readbacks training program. There was no difference in the responses between the surgery and anesthesia departments.

There was a statistically significant difference in the healthcare providers willingness to attend a short training module on readbacks (p < 0.001). Resident physicians were less likely to endorse the importance of readbacks in reducing communication errors (p = 0.01) and less willing to attend a short training module on readbacks (p < 0.001), as compared to staff providers and nursing staff.

The main challenge for respondents, which emanated from their responses, appeared to relate to determining the ideal scenarios in which readbacks may be most appropriately used. Overall, respondents strongly felt that readbacks had an important role in patient handoffs, patient orders regarding critical results, counting and verifying surgical instruments, and delegating multiple perioperative tasks.

**Conclusion:**

The majority of all respondents appear to perceive standardized readbacks as an effective tool for reducing and/or preventing adverse events in the care of surgical patients, derived from a breakdown in communication among perioperative caregivers. Further work needs to be done to define the exact clinical scenarios in which readbacks may be most efficiently implemented, including the definition of a uniform set of scripted quotes and phrases, which should likely be standardized in concert with the aviation safety model.

## Background

Effective communication is a basic human necessity which is particularly critical to assuring safety in high-reliability industries including those involved in the medical and aviation sectors [1,2]. Hundreds of lives can be lost at a time due to a minor error in communication, as demonstrated by catastrophic experiences in civilian aviation.

Several recent air disasters involving miscommunication between crewmembers and air traffic control clearly emphasize that – despite significant advances in civilian aviation safety in recent decades – human errors in judgment and communication still occur, causing devastating consequences. In contrast to the established communication protocols in high risk industries, including commercial aviation, submarine and nuclear technology, surgical safety protocols still fall short of protecting patients from unintended and preventable harm, mainly based on breakdowns in communication among health care providers.

Notwithstanding these recent improvements in surgical safety, a recent analysis by the American College of Surgeons’ closed claims database revealed that a significant source of surgical errors can still be attributed to a breakdown in communication before, during, and after surgery. Breakdowns in verbal communication accounted for around 85 % of adverse events related to communication breakdown, with only 4 % of breakdowns attributed to written communication [3]. As such, the patient safety community has increasingly called for formal readback orders among healthcare professionals who care for surgical patients in order to reduce the high incidence of perioperative complications related to verbal communication breakdowns [1,2,4,5]. The Agency for Healthcare Research and Quality (AHRQ) and the Joint Commission (JC) both recommend the implementation of readbacks in the healthcare setting, particularly during telephone medication orders or verbal transfer of critical tests results [6,7].

While hospitals nationwide have begun to implement readbacks in many of their departments, the use of readbacks is progressing slowly. Dr. Eddie Hoover eloquently described the surgery industry’s reticence to engage in readbacks in a 2007 *Archives of Surgery* editorial when he noted that *“Getting surgeons to readback orders and instructions will age you 10 years, yet the Navies of the world have demonstrated for eons that it improves efficiency, promotes safety, and saves lives.”*[8]

In the past, few trials have been conducted to assess the feasibility and acceptability of surgical safety interventions prior to the intervention itself. The purpose of the current study was to begin to understand the perceptions of and barriers to implementation of readbacks from the point of view of personnel in perioperative services. These data would potentially be used to develop an acceptable and effective local training module, for each category of perioperative personnel, on the appropriate use of readbacks to improve patient safety.

## Methods

This study was based at Denver Health Medical Center, which is Colorado’s principal safety net institution and academic regional level 1 trauma center. The current established patient safety protocols at Denver Health include the Joint Commission’s Universal Protocol [9], standardized “SBAR” communication modules, and the WHO Surgical Safety Checklist [10]. At the time of the study, readbacks were used exclusively for verification of critical test results, in compliance with the Joint Commission’s National Patient Safety Goals [4].

A 12-item questionnaire was designed to evaluate perioperative staff perception of the role of standardized readbacks to improve patient safety in surgery. The specific questions and optional answers are shown in Table 1. A 5-point Likert scale was used for the majority of questions, ranging from “Strongly agree” to “Strongly disagree”. The design of the questionnaire was broadly based on the “Flight Management Attitudes Questionnaire” used to assess risk factors in commercial flight operations [11].

**Table 1:** Overall responses to the survey (n = 92)

	**Strongly Disagree**	**Disagree**	**Neutral / Don’t Know**	**Agree**	**Strongly Agree**
Readbacks in the surgical setting would significantly reduce verbal communication errors and improve patient safety	1 (1.1 %)	3 (3.3 %)	11 (12.0 %)	25 (27.2 %)	52 (56.5 %)
Readbacks are currently being used appropriately by the surgical staff in our hospital	4 (4.4 %)	11 (12.0 %)	17 (18.5 %)	47 (51.1 %)	13 (14.1 %)
I would personally be willing to attend a short training module on readbacks should the concept be formally implemented at my institution	7 (7.6 %)	8 (8.7 %)	15 (16.3 %)	24 (26.1 %)	38 (41.3 %)
*Readbacks would be helpful in reducing verbal communication errors when…*					
… a request is made to carry out an important task that has implications on safety of the patient	1 (1.1 %)	4 (4.4 %)	2 (2.2 %)	25 (27.2 %)	60 (65.2 %)
… there is a handoff of a surgical patient from the care of one provider to another	2 (2.2 %)	2 (2.2 %)	12 (13.0 %)	30 (32.6 %)	46 (50.0 %)
… used to count and verify surgical instruments and other items	1 (1.1 %)	2 (2.2 %)	10 (10.9 %)	27 (29.4 %)	52 (56.5 %)
… there are multiple perioperative tasks	0 (0 %)	4 (4.4 %)	14 (15.2 %)	29 (31.5 %)	45 (48.9 %)
*Significant barriers to implementation of readbacks in the perioperative setting include …*					
… the lack of a general “safety culture” in the surgical department	35 (38.0)	25 (27.2)	13 (14.1)	15 (16.3)	4 (4.4)
… the availability of time to perform readback statements	6 (6.5 %)	17 (18.5 %)	12 (13.0 %)	37 (40.2 %)	20 (21.7 %)
… general reluctance of parts of the surgical team to use readbacks	8 (8.7 %)	13 (14.1 %)	27 (29.4 %)	28 (30.4 %)	16 (17.4 %)
… the amount of training for staff that will be needed to implement readbacks	15 (16.3 %)	31 (33.7 %)	27 (29.4 %)	16 (17.4 %)	3 (3.3 %)

The modality of rolling out the survey consisted of sending an e-mail to all staff in perioperative services (n = 180), including staff surgeons, staff anesthesiologists, residents, midlevel providers, and perioperative nursing staff. The text of the invitation outlined the study hypothesis and concept in detail, and emphasized that study participation was anonymous and purely voluntary with no identifiable information collected. The invitation to complete the survey, including the subsequent reminders, were sent from the office of the Director of Orthopaedic Surgery (PFS). No incentive or compensation for participation was offered or provided. The questionnaire was managed through a “SurveyMonkey™ Premium” software package, which was linked to the hospital’s secure intranet system. Three consecutive e-mail reminders were sent out to all 180 potential respondents, and the survey was closed after three months. The study was approved by the Colorado Multiple Institutional Review Board (COMIRB No. 10–0859).

Frequencies and percentages of specific answers were calculated to explore the usefulness of readbacks and the barriers to implementation. For analysis of frequency, “strongly agree” and “agree” were considered as agreement, and “strongly disagree” and “disagree” were treated as disagreement. Due to the ordinal nature of Likert-scaled items, medians and interquartile ranges were used as measures of central tendency. For analysis, the attending providers and midlevel providers, i.e. certified nurse anesthetists (CRNAs), were aggregated into a single provider category. In addition, the scrub technicians and circulating nurses were collapsed into a nursing staff category. Survey results were compared between departments (surgery vs. anesthesia) by Wilcoxon Rank Sum tests, and between staff roles (MD provider, resident, nursing staff), by Kruskal-Wallis tests. Data analysis was performed using SAS Enterprise Guide 4.2 (SAS Institute, Cary, NC). A *P*-value of less than 0.05 was considered statistically significant.

## Results

Of the 180 perioperative healthcare providers invited to participate in this study, 61 individuals responded after the first invitation, with an additional 31 providers responding after two subsequent reminders. A total of 92 individuals (50.1 %) completed and returned the survey. Of these, 26 (28 %) were attending surgeons, 24 (26 %) were surgical residents, six each were anesthesia attendings (7 %) and anesthesia residents (7 %), 15 (16 %) were circulating nurses, 12 (13 %) were CRNAs, and three (3 %) were surgical scrub technicians. There were no responses from scrub nurses or from registered nurse assistants. Table 2 demonstrates the survey response rate by perioperative staff role. Surgeons had the highest response rate of 70.4 %, with perioperative nursing/scrub techs and anesthesiologists having response rates of 40 % and 37.5 % respectively.

**Table 2:** **Survey responses rates stratified by perioperative staff role**^**#**^

**Perioperative Role**	**Number Queried**	**Number Responded**	**Response Rate**
Anesthesiologists	32	12	37.5 %
Surgeons	71	50	70.4 %
Perioperative Nursing/ scrub techs	75	30	40 %
TOTALS	180	92	51.1 %

^#^ Response rates were calculated by provider type without sub-analysis in the categories “anesthesiologists” and “surgeons”. The group of “perioperative nursing/scrub techs” included CRNAs (certified registered nurse anesthetists).

**Figure 1:**
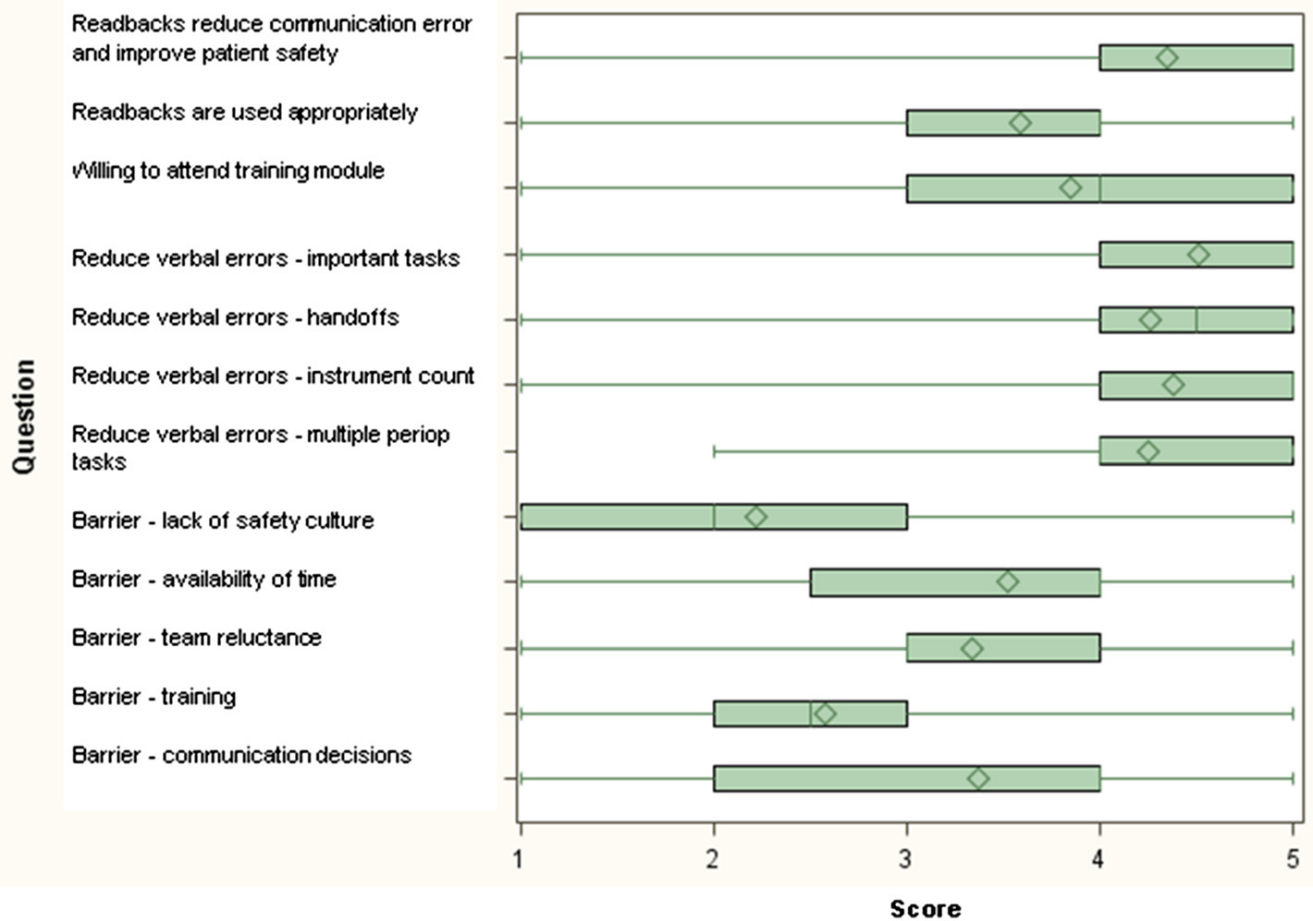
**Box plot depicting the overall survey results.** The length of the box represents the interquartile range (the distance between the 25^th^ and 75^th^ percentiles). The diamond in the box interior represents the mean score. The horizontal line in the box interior represents the median score. The vertical lines (whiskers) issuing from the box extend to the minimum and maximum value.

The overall responses provided by the entire study population (n = 92) are depicted in Table 1. In addition, the graph shown in Figure 1 depicts a box plot with interquartile ranges of all survey responses, for better overview. The majority of participants (83.7 %) agreed that readbacks would significantly reduce verbal communication errors and improve patient safety. However,16.3 % of respondents declared that they would not be willing to attend a short training module on the concept of readbacks, prior to implementation (16.3 %). A majority of participants (65.2 %) disagreed that there was a general lack of a “patient safety culture” within the surgical department.

Table 3 shows the stratification of answers by anesthesia department (n = 24) versus surgery (n = 68). There was no statistically significant difference in the median scores for any response between the two main services (*P* > 0.05). However, significant discrepancies in the perception of the role for readbacks among the different healthcare providers occurred when comparing staffing roles, as outlined in Table 4. On the basis of survey scoring, residents agreed that readbacks significantly reduce verbal communication errors and improve patient safety. Similarly, providers and nursing staff strongly agreed with this statement (p = 0.01). Perioperative nursing staff strongly agreed that they would attend a short training module on readbacks, and staff providers agreed they would attend this course, while residents were neutral regarding the training module (p < 0.001). Nursing staff strongly agreed that the availability of time to perform readback statements was a significant barrier, compared to providers and resident physicians who were less affirmative (p < 0.001).

**Table 3:** **Survey responses stratified by Department**^**#**^

	**Anesthesia n = 24**	**Surgery n = 68**	**P-value**
Readbacks would significantly reduce verbal communication errors and improve patient safety	5 (4–5)	5 (4–5)	0.10
Readbacks are currently being used appropriately by the surgical staff in our hospital	4 (3.5-4)	4 (3–4)	0.77
I would attend a short training module on readbacks should the concept be formally implemented	4 (3–5)	4 (3–5)	0.74
*Readbacks would be helpful in reducing verbal communication errors when …*			
… a request is made to carry out an important task that has implications on safety of the patient	5 (4–5)	5 (4–5)	0.86
… there is a handoff of a surgical patient from the care of one provider to another	4.5 (4–5)	4.5 (4–5)	0.87
… used to count and verify surgical instruments and other items	5 (4–5)	5 (4–5)	0.07
… there are multiple perioperative tasks	4 (4–5)	4.5 (4–5)	0.86
*Significant barriers to implementation of readbacks in the perioperative setting include …*			
… the lack of a general “safety culture” in the surgical department	2 (1–3)	2 (1–3)	0.84
… the availability of time to perform readback statements	4 (3–4)	4 (2–4)	0.54
… general reluctance of parts of the surgical team to use readbacks	4 (3–4)	3 (2–4)	0.07
… the amount of training for staff that will be needed to implement readbacks	2 (2–3)	3 (2–3)	0.13
… the difficulty in deciding what type of communication should constitute a readback	4 (2–4)	4 (2–4)	0.78

^#^ Data are shown as medians and interquartile ranges.

Survey scale:

1 = strongly disagree; 2 = somewhat disagree; 3 = neutral; 4 = somewhat agree; 5 = strongly agree.

**Table 4:** **Survey responses by perioperative staff role**^**#**^

	**Provider n = 44**	**Resident n = 30**	**Nursing Staff n = 18**	**P-value**
Readbacks significantly reduce verbal communication errors and improve patient safety	5 (4–5)	4 (4–5)	5 (5–5)	0.01
Readbacks are currently being used appropriately by the surgical staff in our hospital	4 (3–4)	4 (3–4)	4 (3–5)	0.42
I would attend a short training module on readbacks	4 (4–5)	3 (2–4)	5 (5–5)	<0.001
Readbacks would be helpful in reducing verbal communication errors when …				
… a request is made to carry out an important task that has implications on safety of the patient	5 (4–5)	5 (4–5)	5 (5–5)	0.01
… there is a handoff of a surgical patient from the care of one provider to another	4 (4–5)	4 (4–5)	5 (5–5)	0.12
… used to count and verify surgical instruments and other items	5 (4–5)	4 (3–5)	5 (4–5)	0.08
… there are multiple perioperative tasks	5 (4–5)	4 (4–5)	5 (4–5)	0.41
Significant barriers to implementation of readbacks in the perioperative setting include …				
… the lack of a general “safety culture” in the surgical department	2 (1–3)	2 (1–3)	3 (1–4)	0.14
… the availability of time to perform readback statements	4 (2–4)	4 (2–4)	5 (4–5)	<0.001
… general reluctance of parts of the surgical team to use readbacks	3 (2.5-4)	3 (3–4)	4 (3–5)	0.04
… the amount of training for staff that will be needed to implement readbacks	3 (2–3)	2 (2–3)	3 (2–4)	0.15
… the difficulty in deciding what type of communication should constitute a readback	4 (2–4)	3 (2–4)	4 (3–5)	0.27

^#^The “provider” group includes attending physicians and mid-level providers (CRNAs). The “resident” group refers to physicians in training, while “nursing staff” includes circulating nurses and scrub technicians. Data are shown as medians and interquartile ranges.

Sixty-five percent of the surgical staff felt that readbacks were currently used appropriately, yet 67 % were willing to attend an additional training module on readbacks. Only three participants (3 %) perceived that readbacks would not help reduce errors related to surgical instrument counts. The most significant barrier to readback implementation was the availability of time to perform readback statements (62 %).

## Discussion

The influence of aviation practices on healthcare and patient safety cannot be ignored, whether it be through development of novel surgical safety checklists, skills simulators, and team training. Studies such as those carried out by Gore and colleagues demonstrate the impact of aviation personnel have had on helping to improve the perioperative communication climate and the development of aviation-based perioperative briefings [12]. Indeed, the analogy between aviation and surgery is certainly not new, and several initiatives have evolved to improve perioperative communication and reduce surgical errors [5,13]. These include the implementation of a standardized surgical “time-out” to ensure correct patient identity and correct surgical site, introduction of medical team training programs and of structured perioperative briefings and surgical checklists [9,12,14-16]. Impressively, the simple use of perioperative briefings has been shown to significantly reduce the incidence of wrong-site surgery [17].

In addition to the landmark WHO Surgical Safety Checklist, the Universal Protocol, endorsed by the Joint Commission, has also played an important role in addressing errors contributing to wrong-site procedures and wrong-patient surgery [9,14]. Medical errors continue to receive increasingly widespread attention, fueled in part by the intermittent and troubling occurrence of highly publicized tragic outcomes ostensibly due to communication errors [18,19]. Leading national organizations have thus exhorted health care providers to adopt systems – engineering approaches – that have irrefutably improved safety in other high-risk industries, such as professional aviation and nuclear power technology [4,20].

In a recent study, we reported that despite its wide-scale implementation, the Universal Protocol has failed to eliminate the “never events” of wrong site and wrong patient errors [21]. In a review of nearly 150 wrong-patient and wrong-site procedures, we found that 100 % of errors resulting in wrong-patient procedures, and nearly 50 % of wrong-site procedures, were the result of a communication breakdown [21]. Based on these disturbing findings, we made a strong case for the use of mandatory standardized readbacks, briefings/debriefings, and surgical checklists, in addition to the strict adherence to the Universal Protocol [9,21]. In response to our study, Adelman and Chelcun noted that the WHO Surgical Safety Checklist and the concept of medical team training are important adjuncts to the Universal Protocol [22].

While the implementation of readbacks over the span of many decades into standard flight operations has met with great success, the demands placed on verbal communication in aviation leave much room for improvement. Within the framework of readbacks, several issues still present themselves in flight safety trend analysis. Specifically, recurring errors include: (1) incorrect readbacks by pilots and lack of correction by Air Traffic Control; (2) a correct readback followed by an incorrect action in the cockpit; and (3) a correct readback by the pilot, but an incorrect recording of the readback by the controller, leading to subsequent errors [23,24]. As such, the aviation community has continued to push for unambiguous phraseology, the insistence of complete and accurate readbacks by pilots, and the monitoring of all readbacks by air traffic control [23,24].

While situational differences exist between the flight deck and the operating room, there is an obvious benefit of adopting communication principles of aviation to improve patient safety in surgery [25-28]. As such, the results of this preliminary study demonstrate several important factors in developing a formal program for perioperative readback implementation. First and foremost, there appears to be a strong agreement among perioperative staff that readbacks indeed represent an important communication tool in improving perioperative safety. Staff were also overwhelmingly willing to take part in a short training course to implement readbacks seamlessly and effectively. Interestingly, however, a majority of the respondents believed that readbacks were already being used appropriately in the hospital.

Respondents believed that both the training needed to integrate readbacks as well as the potential reluctance of the surgical staff to use readbacks were minor barriers to implementation. The difficulty in implementation seems to revolve around determining what kind of communication would be appropriate given the time constraints. Broadly speaking, respondents believed that readbacks had an important role in patient handoffs, critical patient orders, counting and verifying surgical instruments, and delegating multiple perioperative tasks. While concerns that the use of readbacks may cause excessive perioperative delays are understandable, a variety of studies have demonstrated that communication safety strategies may actually prevent unexpected delays and communication failures. A previous report investigated the use of preoperative briefings, and found that the briefings were associated with a 31 % reduction in unexpected delays and a 19 % reduction in communication breakdowns leading to delays [29]. As such, in further bolstering the communication process, readbacks may further reduce the incidence of unexpected delays.

Of interest is the statistically significant difference in the strength of agreement on the potential for readbacks to improve patient safety between residents, providers, and nursing staff. Just as important is the significant difference in the healthcare provider willingness to attend a short training module on readbacks, with the strongest support for attending a training course coming from the nursing staff. The lower strength of agreement by residents on the patient safety benefits of readbacks and the lower willingness to attend a training module on readbacks may be due to a variety of factors. The training demands placed on residents may limit their emphasis and exposure to patient safety initiatives, as compared with staff nurses, technicians, and providers. A recent study that reviewed resident engagement in quality improvement initiatives noted several barriers to participation [30]. These barriers included academic medical centers placing a higher value on individual autonomy rather than on commitment to total quality improvement, “resistance to process standardization, and low regard for systems thinking” [30]. The authors also noted that academic centers do not value quality improvement as an academic discipline to the same extent that laboratory or clinical research is valued in career development [30]. As such, residents may be less inclined to commit to quality improvement initiatives.

A recent paper describing a “patient safety curriculum for medical residents” found that residents were frequently not aware of the risks associated with the procedures they carried out and were often unclear on their role in improving patient safety [31]. Indeed, these findings further bolster the need for resident-specific educational programs and opportunities for involvement in critical patient safety initiatives. Another recent study documented that resident-attending intraoperative communication can play a valuable role in preventing adverse patient events further reinforces the importance of securing resident support in the deployment of perioperative readbacks [32]. Moreover, previous work has raised a concerning skepticism that exists among physicians about the value of certain interventions to improve patient safety [33].

An assessment of the “patient safety climate” in 92 hospitals found significant differences in attitudes and perceptions of patient safety and patient safety initiatives amongst different hospital work areas and disciplines, particularly between nurses and physicians [34]. These findings underscore the importance of developing patient safety educational modules that take into account the baseline differences in safety climate between different members of the perioperative staff. Unbridled skepticism will perniciously impede the adoption of new patient safety interventions and thus needs to be investigated and addressed [35].

In a landmark article, Dunn and colleagues reported the successful implementation of a “Medical Team Training Program” derived from the concept of crew resource management in professional aviation [36]. The study outlined the program’s benefit outside of the operating room, e.g. in the settings of critical care interdisciplinary rounds, and clinical unit administrative briefings. Most importantly, the authors emphasized the importance of institutional leadership support for achieving a “critical mass” of staff attendance for training sessions and successful program implementation [36]. Impressively, Neily and colleagues demonstrated in a more recent follow-up study that the participation in the same Medical Team Training program was associated with lower surgical mortality rates [16]. The authors showed that – for every quarter of the training program – a reduction of 0.5 deaths per 1,000 surgical procedures was achieved [16].

Limitations of this preliminary study include the moderate survey return rate of 50.1 %, leading to a relatively small sample size of n = 92. The limited sample size may be one of the contributing factors to the difficulty in determining respondent trends as a function of their perioperative role. Additionally, the distribution of perioperative staff was heavily skewed towards surgical residents and attending surgeons, with fewer respondents coming from the anesthesia and nursing departments. As such, broadening the sample size to include a greater number of respondents from the latter two departments and combining data from other institutions will increase the generalizability of the results. Further enquiries may also be conducted through observing response patterns as a function of whether the respondents work at an academic or community hospital. Indeed, observing and appreciating attitudinal pattern variability would be critical in developing tailored readback programs that would be acceptable to different departments in terms of content delivery and didactics. To assure greater participation from low responding provider types, it may beneficial to approach department heads and request that they disseminate the survey to their staff and strongly encourage them to complete the survey. This may provide more incentive and be more beneficial that sending the survey to staff members from a centralized and non-affiliated research group.

In terms of improving the survey in future studies, it may be beneficial to provide more detailed scenarios to the respondent where the perceived utility of readbacks could be queried. Furthermore, in order to determine the baseline use of readbacks, respondents could be queried on 1) whether they believe that they are using readbacks appropriately, and 2) whether other perioperative staff are using readbacks appropriately. This may provide provider-specific baseline data on readback use as well as further perceptions on teamwork between perioperative staff categories.

Most importantly, developing a list of specific items and scenarios where readbacks would confer the most benefit is critical to the development of a training module. As demonstrated in the results, the difficulty in determining what is appropriate to be read back in all perioperative settings stands as one of the greatest challenges in the development and implementation of a specific curriculum. In this regard, Guise and colleagues developed a “Clinical Teamwork Scale” to evaluate clinical teamwork skills based on simulation exercises and in everyday clinical care [37]. The ease of applicability and reliability of the scale makes it a useful prospective validation tool for evaluation of the quality of a future readback program [37].

## Conclusions

While an expanded sample size for the survey study would be beneficial in detecting greater response variations in attitudes towards readbacks as a function of provider and institutional type, this preliminary study provides some support for the development of readback training programs to improve closed-loop communication and patient safety. However, much work needs to be done in determining an appropriate list of specific tasks and scenarios where readbacks would be most helpful in preventing communication errors. Additionally, department-specific focus groups would be of benefit in further querying reasons why certain parts of the surgical team would be reluctant to integrate readbacks into their communication strategy. It is encouraging to know, however, that perioperative providers recognize the usefulness of this aviation-based communication strategy, and moreover, are willing to actively participate in its implementation. Beyond a doubt, the implementation of such a proactive new protocol can only be accomplished with unlimited institutional leadership support.

## Competing interests

The authors declare no competing interests related to this study.

## Authors’ contributions

HP, JC and PFS designed the study protocol and the survey questionnaire. PFS and PSM implemented the survey at Denver Health, and assisted with analysis of the data and interpretation of the results. AS performed the statistical analysis of the data. HP wrote the first draft of the manuscript. AS, PFS, SW, and JC revised the manuscript into final form. All authors contributed to revisions to the manuscript, and read and approved the final version.

## Pre-publication history

The pre-publication history for this paper can be accessed here:

http://www.biomedcentral.com/1471-2482/12/8/prepub
